# Family socio-economic status and parental education anxiety: the mediating role of perception of the “double reduction” policy and the moderating role of parental education expectations

**DOI:** 10.3389/fpsyt.2025.1525651

**Published:** 2025-06-02

**Authors:** Jinfang Niu, Jie Fang, Zhanyong Qi

**Affiliations:** ^1^ Faculty of Education Sciences, Henan Finance University, Zhengzhou, China; ^2^ Faculty of Education, East China Normal University, Shanghai, China; ^3^ Faculty of Education, Shaanxi Normal University, Xi’an, China

**Keywords:** compulsory education in China, the “double reduction” policy, parental education anxiety, family socio-economic status, parental education expectations, perceived the “Double Reduction” policy, mediating effect, moderating effect

## Abstract

**Background:**

The “Double Reduction” policy promulgated by the Chinese government in 2021 aims to alleviate parental education anxiety as one of the policy goals, blocking the path of parents’ high participation in their children’s education through intensive out-of-school training and a large number of homework exercises in the past, to comprehensively promote educational equity. However, with the continuous promotion of the policy since its initial introduction, it now faces new controversies. Parents from varying family socio-economic backgrounds exhibit divergent responses to the “Double Reduction” policy, necessitating an examination of parental education anxiety through the lens of family socio-economic status at this juncture.

**Methods:**

A survey involving 2,932 parents in China was conducted utilizing the Family Socioeconomic Status Scale, Parents Educational Anxiety Scale, Perception of the “Double Reduction” Policy Scale, and Educational Expectations Scale.

**Results:**

The findings reveal several key insights: first, educational anxiety is prevalent among parents with an overall medium-to-high intensity level; notably, academic attitude anxiety is most pronounced; second, for each unit increase in a family socio-economic status, there is a corresponding decrease of 0.062 in parental educational anxiety; third, perception of the “Double Reduction” policy effectiveness partially mediates the relationship between family socio-economic status and educational anxiety, while the understanding of the “Double Reduction” policy does not serve as a mediating factor; fourth, parental educational expectations moderated the relationship between perception of the “Double Reduction” policy effectiveness and educational anxiety.

**Discussion:**

Four years post-implementation of this policy, and the re-evaluation of the real situation of parents’ education anxiety helps to deeply understand the implementation effect of the “Double Reduction” policy, so as to provide a reference direction for the detailed adjustment of the policy.

## 1 Introduction

In July 2021, the General Office of the Central Committee of the CPC and the General Office of the State Council issued the Opinions on Further Reducing the Burden of Homework for Students in Compulsory Education and the Burden of Out-of-School Training (hereinafter referred to as the “Double Reduction” policy), to alleviate the group parental education anxiety triggered by the massive entry of capital by reducing both the total amount of students’ in-school work and the burden of out-of-school training, and to reshape the position of schools as the main position of education. The “Double Reduction” policy has attracted widespread attention from all sectors of the community since its promulgation, and has been in effect for more than three years now, achieving great results while facing numerous controversies (1). On the one hand, the effectiveness of the radical “Double Reduction” policy, which requires a complete elimination of after-school tutoring institutions, has gradually been highly questioned by different scientists (2, 3); On the other hand, parents and other educational subjects out of their respective interests (4) and take a series of practices contrary to the original intent of the policy, forming a forming a contradictory situation of “reducing the burden on students in school and increasing the burden on parents invisibly”.

Parents in different classes adopt differentiated attitudes and response measures to deal with the “Double Reduction” policy (5). The “Equal Opportunity in Education report” authored under the auspices of Coleman, identified the families socio-economic status (SES) as the primary factor in the creation of educational inequality (6). According to family investment theory, families SES directly determines the extent of resources available to parents (7), and there is a positive correlation between family capital and children’s access to education (8). Differences in family conditions will aggravate the gap in educational results (9). Inequality in the possession of resources exacerbates PEA.

PEA directly determines whether the “Double Reduction” policy can be sustained (10). The new controversy facing the “double reduction” policy and the PEA of different families SES need to be further examined. Drawing on survey data collected from Chinese households, this study examines how family socioeconomic status shapes parental education anxiety under the “Double Reduction” policy framework, analyzing both its current manifestations and underlying mechanisms. This study not only helps to explain the reasons why parents with different family SES adopt different responses to the “Double Reduction” policy, but also helps to expose the complex relationship between the policy implementation effect and family choices, and provides empirical data to support the refinement and adjustment of the “Double Reduction” policy in the next stage.

## 2 Literature review

### 2.1 Parental education anxiety in the context of the “Double Reduction” policy

Anxiety refers to physiological symptoms such as increased heart rate, muscle tension and shortness of breath when individuals face unpleasant threats (11). PEA is a concrete reaction of anxiety in the field of education, different studies have different interpretations of the connotation of PEA, but they often misappropriate the definition of “anxiety” in the field of psychology to define it conceptually, narrowing PEA into a set of negative emotions caused by parents’ non-determinism of educational process and educational results (12). However, in fact, PEA in the context of the “Double Reduction” policy has taken on new features. First of all, students’ academic performance remains the primary focus of parental attention (13). Within the context of future academic assessments continuing to be predicated on grades, the diminishment of students’ homework assignments and examination frequency engenders widespread parental concern. Specifically, parents fear that diminished practice opportunities may lead to deterioration in their children’s test-taking proficiency, thereby potentially compromising academic competitiveness (14); Secondly, the “Double Reduction” policy is mainly aimed at primary and secondary schools, but students still have to face the pressure of entering high schools and colleges (15), and a study has shown that PEA about choosing schools in the context of the “Double Reduction” policy has not been alleviated (16); Thirdly, parents are concerned that students’ attitudes towards learning are lax due to the reduction of homework training and the lack of external incentives for academic performance; Finally, the “Double Reduction” policy puts forward higher requirements for parents’ upbringing ability. Parents can no longer rely on the intervention of out-of-school counseling agencies to provide additional counseling for their children as in the past (17). Based on this, the first hypothesis of this research is proposed:

Hypothesis 1 (H1): Parental education anxiety is still common in the context of the “Double Reduction” policy.

### 2.2 Family socio-economic status and perception of the “Double Reduction” policy influence parental education anxiety

#### 2.2.1 Family socio-economic status influences parental education anxiety in the context of the “Double Reduction” policy

Family SES refers to the hierarchical ranking of families based on the valuable resources they can control (18), and is a derivative concept developed by the German sociologist Weber based on the theory of social stratification (19). In the field of sociology, ascribed resources are considered to be the sum of resources that an individual can acquire automatically without any acquired effort due to innate factors (20), and family SES is a type of ascribed resources (21). Pierre Bourdieu’s cultural capital theory divides family SES into three dimensions: economic capital, social capital and cultural capital (22). Among them, economic capital is the financial assets with currency as the main form; Social capital is the explicit or invisible network of social relations. Cultural capital is the educational qualifications and academic diplomas acquired in the institutionalized education system (23). Parents with disadvantaged family SES have less economic, social and cultural capital, which leads to their weak adaptability (24), and PEA is constantly catalyzed in the process of trying to help their children achieve class transition (25). Simultaneously, when the power distribution within a group is imbalanced, parents from dominant family backgrounds often experience anxiety due to their awareness that the prevailing inequality will inevitably shift (26). Although the “Double Reduction” policy explicitly prohibits after-school training institutions, parents with an advantageous family SES can still provide additional educational opportunities for their children by spending large sums of money privately and hiring one-on-one tutors (27), which will further widen the education gap. Therefore, the second hypothesis of this study is proposed:

Hypothesis 2 (H2): Family socio-economic status has a significant negative effect on parental education anxiety, parents with better family socio-economic status are less likely to be anxious.

#### 2.2.2 Perception of the “Double Reduction” policy affects parental education anxiety

Perception mainly refers to the subjective feeling process of the individual to the objective things (28). Policy perception is the subjective feeling of the policy object to the policy text and the implementation content, and it is the direct reaction of the policy implementation effect (29). Parents’ perception of the “Double Reduction” policy (PDRP) comprises two dimensions: understanding of the “Double Reduction” policy (UDRP) and perception of the “Double Reduction” policy effectiveness (PDRPE) (30). Individual perception plays a core leading role in shaping behavior (31). The allocation of resources prescribed by policies shapes the behavior of target groups by influencing their perceptions (32). There is a huge cognitive difference between policy makers and target groups (33), which leads to “unavoidable conflicts” (34). Parents, as important participants of the “Double Reduction” policy, often blindly pursue short-term interests and respond to the policy rashly, resulting in the neglect of the long-term interests of education (35). A study shows that the more deeply parents perceive the policy, the more they recognize the value of the policy (36). However, there is also a study that shows a negative relationship between PDRP and PEA. Therefore, the third hypothesis of this study is proposed:

Hypothesis 3 (H3): parents’ perception of the “Double Reduction” policy has a significant negative impact on parental education anxiety, with greater understanding of the “Double Reduction” policy and perception of the “Double Reduction” policy effectiveness alleviating such anxiety.

### 2.3 Family socio-economic status affects parental education anxiety through perception of the “Double Reduction” policy

#### 2.3.1 Family socio-economic status directly affects perception of the “Double Reduction” policy

Individuals will perceive and evaluate stressors based on their own resources (37). According to the family investment theory, parents with superior family SES can participate in education with scientific educational theories and methods by virtue of their higher economic and cultural capital (38), so as to face the changes in education brought by the “Double Reduction” policy more calmly. Parents with disadvantaged family SES are powerless in the face of the new “Double Reduction” policy (39), and are under long-term mental pressure due to the lack of their own economic, social, and cultural resources. Accordingly, the fourth research hypothesis of this study is proposed:

Hypothesis 4 (H4): Family socio-economic status has a significant positive impact on perception of the “Double Reduction” policy, parents with better family socio-economic status have a deeper understanding of the “double reduction” policy and higher perception of the “Double Reduction” policy effectiveness.

#### 2.3.2 Family socio-economic status affects parental education anxiety through perception of the “Double Reduction” policy

American psychologist Bandura proposed the three-way interaction determinism, which holds that the three factors of an individual’s environment, perception and behavior are closely related and are always in dynamic interaction (40). The individuals’ potential behavior tendency can be transformed into actual behavior in the environment, and perceptual factors play a decisive role in this process. As a proxy variable of family environment, family SES has a significant impact on PEA. In this process, parents’ PDRP affects the internalization of the influence of family SES and the generation of PEA. Therefore, the fifth hypothesis of this study is proposed:

Hypothesis 5 (H5): Perception of the “Double Reduction” policy plays a significant mediating role in the influence of family socio-economic status on Parental education anxiety.

### 2.4 The moderating role of parental education expectations

Parental education expectations(PEE) is the expectation of parents for the level of education that their children can finally receive. (41) In the Chinese cultural symbol system, the “dragon” symbolizes imperial power, strength, and auspiciousness, mostly representing the masculine spirit and extraordinary abilities of males; the “phoenix” embodies auspiciousness, virtue, and nobility, mainly symbolizing the top status and complete achievements of females. Both are core totems of Chinese civilization, carrying an idealized projection of perfect personality and social status. The traditional Chinese thought of “hoping children will become dragons and hoping girls will become phoenixes” expresses the high PEE Chinese parents have for their children, emphasizing achieving social class leap through education and demanding that children become social elites (42). However, PEE as “significant others’ encouragement” (43), too high degree is not conducive to the achievement of the established educational goals. Parents hope their children can achieve higher academic performance in the fairer educational environment created by the “Double Reduction” policy. When the gap between children’s objective learning results and subjective expectations is too large, parents will feel educational anxiety. Based on this, the sixth hypothesis of this study is proposed:

Hypothesis 6 (H6): Parental education expectations moderates the effect of perception of the “Double Reduction” policy on parental education anxiety.

**Figure 1:**
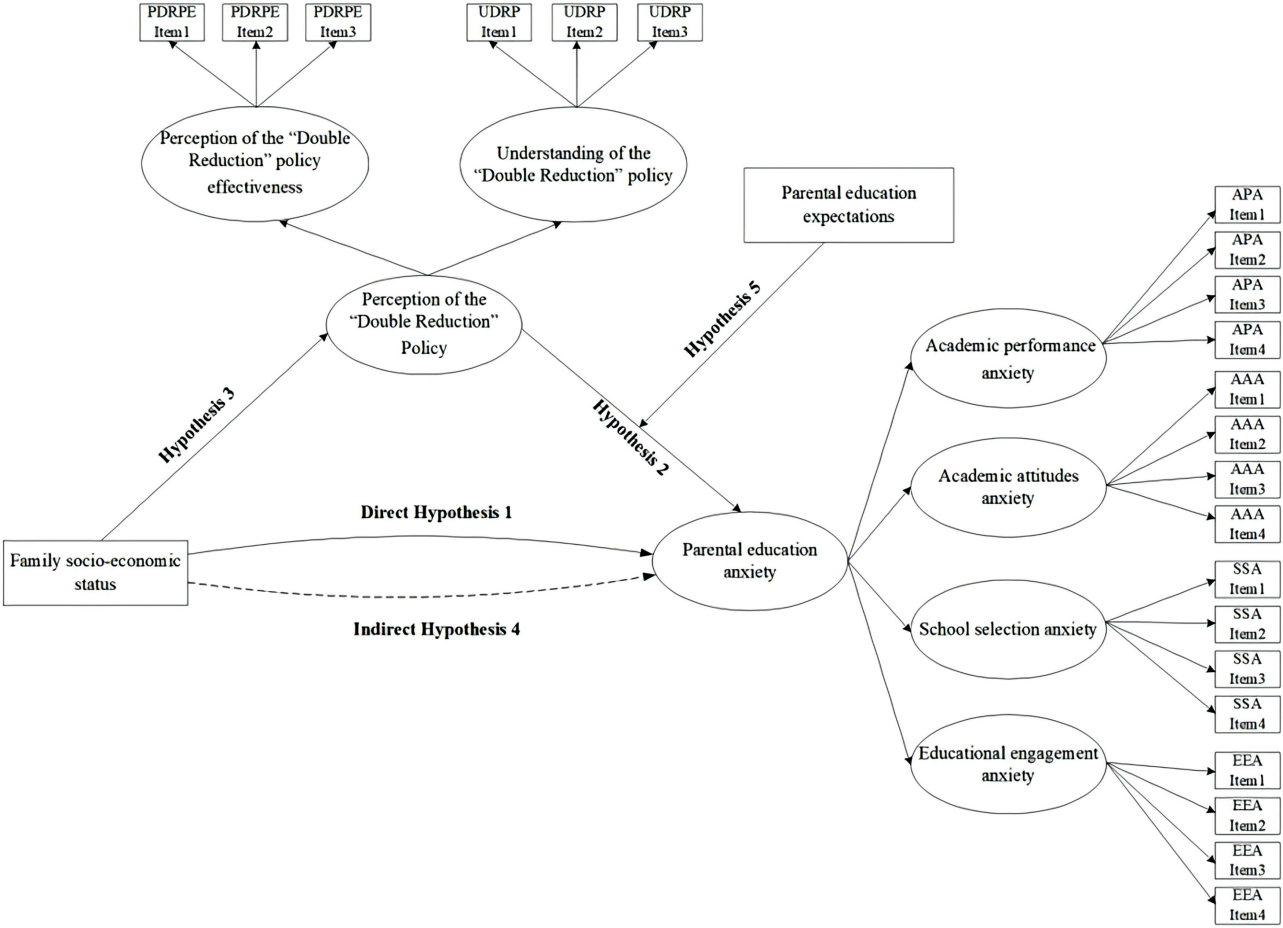
Theoretical framework (The arrows indicate the direction of influence, the solid lines represent direct influence, and the dashed lines represent indirect influence).

To summarize, current research on the “Double Reduction” policy exhibits two main limitations: First, existing literature predominantly focuses on policy text analysis while lacking empirical examination of parental education anxiety; Second, systematic analyses of policy implementation effects from the family SES perspective remain particularly scarce. To address these critical questions, this study employs the Family Socio-economic Status Scale, Parental Education Anxiety Scale, Perception of the “Double Reduction” Policy Scale, and Parental Education Expectations Scale to verify six research hypotheses reflecting complex inter-variable relationships, specifically: Parental education anxiety persists three years after the implementation of the “Double Reduction” policy (H1); Parents with higher family socio-economic status exhibit lower anxiety levels (H2); Parental perception of the “Double Reduction” policy negatively influences parental education anxiety (H3); Family socio-economic status positively influences perception of the “Double Reduction” policy (H4); Perception of the “Double Reduction” policy plays a significant mediating role in the influence of family socio-economic status on parental education anxiety (H5); Parental education expectations moderates the effect of perception of the “Double Reduction” policy on parental education anxiety (H6). The marginal contribution of this study resides in establishing a mediating model (see **Figure 1** below) that adopts parental perspectives as the analytical lens to investigate the implementation outcomes of the “Double Reduction” policy. By elucidating the complex interplay among family SES, perceptions of the “Double Reduction” policy, and educational expectation levels, this research fills a critical void in understanding the pathways influencing parental educational anxiety within the “Double Reduction” policy context.

## 3 Methods

### 3.1 Data sources and sampling methods

This study investigated the education status of families in the compulsory education stage in China from June 2024 to September 2024. This study employed online questionnaire administration based on two methodological rationales. First, given the substantial sample size (N>10,000), traditional paper-based distribution among parental populations would impose prohibitive costs (including human resources, temporal expenditure, and material logistics). Second, face-to-face data collection might induce social desirability bias that compromises response authenticity. The electronic questionnaire system addressed these limitations through dual mechanisms: cost-effective data acquisition covering eight strategically selected provincial-level regions across eastern, central, and western China, coupled with anonymization protocols to mitigate social expectation effects. Ethical compliance was ensured through a dual verification framework: 1) mandatory review and electronic signing of informed consent documents (e-ICF), and 2) technical barriers preventing access to core questionnaire modules without completed consent verification. This operational design adheres to the ethical standards for social science research outlined in the Declaration of Helsinki, while maintaining procedural rigor through three implementation safeguards: geolocation validation of respondents, real-time response consistency checks, and encrypted data transmission protocols. The methodological architecture balances ecological validity with administrative feasibility in large-scale parental studies.

The main reasons for focusing the study on the compulsory education stage (CES) are as follows: first, the education stage targeted by the “Double Reduction” policy is compulsory education stage (stipulated in the Education Law of China that a total of nine years from the first grade of primary school to the third grade of junior middle school is CES); second, the education diversion problem after the CES in China is a crucial starting point for the differentiated development of students educational careers. The level and type of high school students enter after this will directly determine their level of higher education, which makes PEA highlighted in the CES; third, compared with the high school stage, students in the CES have less academic difficulty, the threshold for parents to participate in their children’s studies is weakened, and their subjective willingness is stronger.

This study adopted a combination of random sampling and stratified sampling to select the sample: First, 8 representative provinces (municipalities) in western, central, and eastern China were selected according to the characteristics of economic development; second, the provincial capitals, cities (at the prefecture level and county level), and towns were selected in each province; third, one primary school and one middle school were randomly selected in each city/town, and at least two families were selected in each grade; fourth, the principal of each school sent the questionnaire to parents via the “Wenjuanxing” (data collection website) by providing a web link. A total of 3200 questionnaires were distributed and 2932 were recovered, with a recovery rate of 91.63%. Considering that the response time is obviously too short to be in line with common sense, and the answers are vacant, skip and wrong, a total of 2865 valid questionnaires remain after deleting invalid questionnaires, with a valid rate of 89.56%. Descriptive statistics of respondents are shown in **Table 1** below.

**Table 1:** Demographics distribution of samples (N = 2865).

Demographic variables	Category	Frequency	Percentage	Demographic variables	Category	Frequency	Percentage
**Gender**	Male	732	25.5%	**Gender of children**	Male	1510	52.7%
	Female	2133	74.5%		Female	1355	47.3%
**Age**	<30 years	81	2.8%	**Children's grades**	Excellent	275	9.6%
	31-40 years	1758	61.4%		Good	799	27.9%
	41-50 years	947	33.1%		Middle	981	34.2%
	51-60 years	65	2.3%		Pass	466	16.3%
	≥60 years	14	0.5%		Failure	344	12.0%
**Educational expectation**	Stop studying	2	0.1%	**Political status**	Communist	238	8.3%
	Primary school	13	0.5%		Communist Youth League	257	9.0%
	Junior high school	44	1.5%				
	High school	91	3.2%		Democratic parties	7	0.2%
	Junior college	80	2.8%		Mass	2363	82.5%
	Undergraduate course	930	32.5%	**Only child**	Yes	596	20.8%
	Master’s or doctoral students	1705	59.5%		No	2269	79.2%
**School Location**	City	1390	48.5%	**School Attributes**	Public School	2609	91.1%
	Rural	1475	51.5%		Private School	256	8.9%

Gender, the gender of the parent; Age, the age of the parent; Educational expectation, the highest level of education the parent hopes their child will achieve; School Location, the physical location of the child's school; Political status, the political party or group the parent is affiliated with; Only child: whether there is only one child in the family; School Attributes, the ownership of the child's school.

### 3.2 Description of variables

In this research, all the scales employed are mature and validated both domestically and internationally. Taking into account the specificity of the “Double Reduction” policy, the final survey questionnaire was optimized based on parental feedback gathered from an initial pre-survey. The specific items of the questionnaire are detailed in **Table 2**. In addition to the control variables, the questionnaire adopts the Likert five-point scale, ranging from “1” (indicating “Very Disagree”) to “5” (denoting “Very Agree”), to represent a gradual transition from disagreement to agreement.

**Table 2:** Total questionnaire specific psychometric information (N = 2865).

Questionnaire	Dimensions	Title items	Number of items
Background		Gender; Age; Gender of children; Children’s grades; School Location; Only child and school affiliation	7
Parental education anxiety	Academic performance anxiety	APA1: In general, I feel anxious about my child's academic performance	16
		APA2: After the “double reduction”, I worry that the reduction of homework and exams will reduce students' academic competitiveness.	
		APA3: I feel nervous when I hear that my children are going to have exams.	
		APA4: I was nervous before checking my children's rankings.	
	Academic attitudes anxiety	AAA1: I am worried about my children's lack of interest in learning.	
		AAA2: I am worried about my children's lack of initiative, unconsciousness and initiative in their studies.	
		AAA3: I am worried that my children are afraid of their studies, and they are easy to quit.	
		AAA4: I am very worried that my children will lose their will and neglect their studies because of mobile phones and other electronic products.	
	School selection anxiety	SSA1: I'm worried about not having a school district room.	
		SSA2: I was torn and worried about choosing a school for my children.	
		SSA3: I am very worried that there is no access to detailed information about each school.	
		SSA4: I am concerned that there is no proper channel to understand the admission policies of each school.	
	Educational engagement anxiety	EEA1: I am very worried that I do not have the ability to help my children with their schoolwork.	
		EEA2: I am very worried I didn't have a scientific upbringing.	
		EEA3: I am very worried that I can not handle the parent-child relationship well when tutoring children.	
		EEA4: I am worried that my children will resent my excessive involvement in school.	
Perception of the “Double Reduction” policy	perception of the “Double Reduction” policy effectiveness	PDRPE1: I understand the text of the “double reduction” policy.	6
		PDRPE2: I understand the policy objectives of the “double reduction” policy.	
		PDRPE3: I understand the implementation rules and implementation of the “double reduction” policy in schools.	
	Understanding of the “Double Reduction” policy	UDRP1: I think the “double reduction” policy has reduced the burden of school work for children.	
		UDRP2: I think the “double reduction” policy has reduced the burden of out-of-school training for children.	
		UDRP3: In general, I agree with the “double reduction” policy.	

#### 3.2.1 Independent variable: family socio-economic status

In the context of the PISA international assessment (44), the socioeconomic status of families is evaluated through three key indicators: familial cultural capital, operationalized as the highest educational attainment of the parents; familial social capital, represented by the highest occupational tier occupied by the parents; and familial economic capital, measured by the household’s per capita income. Concurrently, Scientists (45, 46) widely employ the principal component analysis (PCA) to synthesize this three indicators into a composite measure representing the family SES. The PCA method fundamentally aims to maximize the explained variance of the original data by generating a set of uncorrelated principal components through linear combinations, thereby optimally preserving data variability. This intrinsic characteristic renders PCA particularly suitable for synthesizing composite indicators. In contrast, factor analysis (FA) primarily focuses on explaining covariance among variables, making it more applicable for exploring latent theoretical constructs between variables (e.g., scale development) rather than mere data compression or index aggregation. Consequently, this study adopts PCA for SES aggregation.

Specifically, the highest level of education attained by one of the parents ranges from “no schooling” to “master’s or doctoral degree”, corresponding to a total of seven options. These options are assigned values on a scale from low to high, with higher scores indicating a higher level of family socio-cultural capital.

In accordance with the “Chinese Occupational Standards”, the highest occupational tier engaged in by parents is categorized into five distinct types, with each type corresponding to a numerical value ranging from 1 to 5. A higher score signifies a higher level of occupational hierarchy: (1) Temporary Workers, Unemployed Persons, and Agricultural Laborers; (2) Manual Laborers, Self-Employed Individuals, Technical Practitioners, and Equivalent Workers (e.g., Construction Workers); (3) Managers, Technical Staff, and Administrative Personnel (e.g., Sales Associates); (4) Middle Managers, Intermediate Technical Experts, and Various Professionals Employed in Diverse Economic Units (e.g., Lawyers, Educators); (5) Senior Executives, High-Level Technical Leaders, and Senior Managers with Significant Authority in Government Departments, Public Institutions, and Social Organizations (e.g., Civil Servants, Corporate Managers).

The National Bureau of Statistics of China releases annual data on the disposable income of residents for the previous year in the second half of each calendar year. It ranks all surveyed households based on their per capita income from highest to lowest and divides them into five equal quintiles. This study adopts this approach by classifying households in the lowest 20% of per capita income as the low-income group, followed by the lower-middle-income group, the middle-income group, the upper-middle-income group, and finally, the high-income group. Each of these quintiles corresponds to a score ranging from 1 to 5, with higher scores indicating a higher level of household economic capital.

The PCA results indicate that only the first component exhibits an eigenvalue exceeding 1, cumulatively explaining 59.03% of the total variance (see **Tables 3**, **4**) Within the surveyed sample, family SES values range from -2.43 to 3.73, demonstrating characteristics consistent with a standardized normal distribution.

**Table 3:** Interpretation rate of variance in principal component analysis of family SES.

Component	Initial eigenvalue			Extract the sum of squared loads		
	Total	Variance percentage	Accumulated /%	Total	Variance percentage	Accumulated /%
**1**	1.771	59.03	59.03	1.771	59.03	59.03
**2**	0.834	27.803	86.834			
**3**	0.395	13.166	100			

Extraction method is Principal Component Analysis.

**Table 4:** Principal component analysis factor loadings for family SES.

Component	1
	Factor loading
highest educational attainment of parents	0.851
highest occupational status of parents	0.856
percapita household income	0.560

Extraction method is Principal Component Analysis; Extracted 1 principal component.

#### 3.2.2 Dependent variable: parental education anxiety

Building upon a comprehensive review of both domestic and international research literature on PEA, and considering the novel manifestations of PEA in the wake of the “Double Reduction” policy, this study utilizes a self-designed Parental Educational Anxiety Scale, consisting of a total of 16 items, to assess the level of PEA, and the specific items of the scale are presented in **Table 2**. The scale is structured around four factors: Academic Performance Anxiety (APA, with 4 items), Academic Attitude Anxiety (AAA, with 4 items), School Selection Anxiety (SSA, with 4 items), and Educational Engagement Anxiety (EEA, with 4 items). The final score obtained from the parental questionnaire is directly proportional to the intensity of their PEA, indicating that higher scores signify a greater degree of anxiety. The results of the internal consistency analysis revealed Cronbach’s α coefficients of 0.802, 0.880, 0.819, 0.898, and 0.961 for the four factors and the overall scale, respectively. All coefficients exceeded the threshold of 0.8, indicating a reasonable level of internal consistency reliability for the scale. The results of the exploratory factor analysis (EFA) reveal that the KMO value for the scale is 0.930, and the p-value corresponding to the Bartlett’s Test of Sphericity is statistically significant at the 0.001 level. In this study, all scales were self-assessed by parents, necessitating the use of Average Variance Extracted (AVE) and Composite Reliability (CR) values to assess the reliability of both the overall scale and its internal dimensions. The results, as presented in **Table 5**, demonstrate that the reliability of the parent self-assessment scale meets the required standards. The results of the confirmatory factor analysis (CFA), as shown in **Table 6**, indicate that the scale’s fit indices all meet the required standards, suggesting good structural validity. In conclusion, the PEA scale developed in this study demonstrates good validity and reliability.

**Table 5:** Convergent validity of measuring models (N = 2865).

Scale	Dimension	Item	Estimate	AVE	CR
PEA	APA	APA1	0.822	0.534	0.814
		APA2	0.781		
		APA3	0.752		
		APA4	0.720		
	AAA	AAA1	0.804	0.662	0.886
		AAA2	0.785		
		AAA3	0.739		
		AAA4	0.725		
	SSA	SSA1	0.808	0.551	0.828
		SSA2	0.804		
		SSA3	0.662		
		SSA4	0.627		
	EEA	EEA1	0.743	0.691	0.900
		EEA2	0.742		
		EEA3	0.733		
		EEA4	0.631		
PDRP	PDRPE	PEDRP1	0.893	0.700	
		PEDRP2	0.890		
		PEDRP3	0.822		
	UDRP	UDRP1	0.852	0.800	
		UDRP2	0.794		
		UDRP3	0.787		

PEA, Parental Education Anxiety; APA, Academic Performance Anxiety; AAA, Academic Attitudes Anxiety; SSA, School Selection Anxiety; EEA, Educational Engagement Anxiety; PDRP, Perception of the “Double Reduction” Policy; PDRPE, Perception of the “Double Reduction” Policy Effectiveness; UDRP, Understanding of the “Double Reduction” Policy.

**Table 6:** Confirmatory factor analysis (CFA) results (N = 2865).

Scale	Fit Indices							
	X²/df	GFI	AGFI	NFI	IFI	TLI	CFI	RMSEA
PEA	11.557	0.951	0.932	0.959	0.962	0.954	0.962	0.061
PDRP	19.001	0.982	0.953	0.986	0.986	0.975	0.986	0.079
Index standard	Large sample	>0.9	>0.9	>0.9	>0.9	>0.9	>0.9	<0.08

PEA, parental education anxiety; PDRP, Perception of the “Double Reduction” Policy. GFI, Goodness of Fit Index; AGFI, Adjusted Goodness of Fit Index; NFI, Normed Fit Index; IFI, incremental fit index; TLI, Tucker Lewis Index; CFI, Comparative fit index; RMSEA, Root mean square error of approximation.

#### 3.2.3 Mediating variable: perception of the “Double Reduction” policy

This study employs the parents’ perception of the “Double Reduction” policy(PDRP)developed by Ding Yadong et al. (30), which is reported by parents and has been demonstrated to possess good reliability and validity in previous research (47). The scale comprises two dimensions: parents’ understanding of the “Double Reduction” policy (UDRP), which assesses their comprehension of the textual content, policy objectives, and implementation details; and their perception of the “Double Reduction” policy effectiveness (PDRPE), which focuses on their perceptions of the policy’s effectiveness in reducing students’ in-school burdens, off-school shadow education burdens, and its overall impact. The detailed items of these dimensions are outlined in **Table 2**. The test results indicate that the internal consistency coefficients for both factors of the scale and the overall scale have consistently surpassed the threshold of 0.8. The KMO measure stands at 0.847, and the Bartlett’s Test of Sphericity yields a statistically significant result at the 0.001 level. Furthermore, two common factors were successfully extracted, collectively accounting for 79.442% of the variance. The specific values for AVE and CR are detailed in **Table 5**. The outcomes of the CFA as presented in **Table 6**, reveal that all scale fit indices have met the anticipated requirements. In conclusion, the PDRP Scale continues to exhibit good validity and reliability in this study.

#### 3.2.4 Moderating variable: parental education expectations

This study investigates PEE for their children through the item “How far do you hope your child will ultimately pursue their education?” It categorizes these expectations into seven options: “Stop schooling now = 1, Primary School = 2, Junior High School = 3, High School or Secondary Vocational School = 4, Polytechnic College = 5, Undergraduate Degree = 6, and Graduate Degree (Master’s or Doctoral) = 7.” A higher numerical value indicates a higher level of PEE held by the parents.

#### 3.2.5 Control variables

Building upon previous research findings, this study incorporates the individual characteristics of the survey participants as control variables, specifically including factors such as the gender, age, political affiliation of the parents, and the gender of the child.

### 3.3 Research methodology

Firstly, data analysis was conducted using SPSS 26.0 and Amos 24.0 software to jointly examine the impact of family socio-economic status on parental education anxiety in the context of the “double reduction” policy, as well as the mediating role of perception of the “Double Reduction” policy and the moderating role of parental education expectations. SPSS 26.0 is used for common method bias testing, descriptive analysis, and correlation analysis; Amos 24.0 was used for confirmatory factor analysis.

Secondly, this study utilizes Model 4 from the SPSS PROCESS program developed by Hayes, in conjunction with the step-by-step method proposed by scientists (48), to test the mediating effect of perception of the “Double Reduction” policy on the influence of family SES on parental education anxiety by constructing Equations 1, 2, 3). Additionally, since both the dependent variables and the mediating variables involved in this study are continuous, Ordinary Least Squares estimation is employed to estimate the regression coefficients in Equations 1-3.


(1) $$Y=\beta_{0}+cSES+\beta_{1}+e_{1}$$

(2) $$M=\alpha_{0}+aSES+\alpha_{1}+e_{2}$$

(3) $$Y=\gamma 0+c'SES+bM+\gamma 1I+e_{3}$$

Y represents parental education anxiety as the dependent variable, family SES as the independent variable, perception of the “Double Reduction” policy as the mediating variable M, I represents the control variable included in this study, and e_1_ represents the random error term. The coefficient a in the above equation represents the influence of X on M, the coefficient b represents the influence of M on Y, the coefficient c’ represents the influence of X on Y through M, and the coefficient c represents the influence of X on Y.

Thirdly, Model 14 from the SPSS PROCESS program authored by Hayes is utilized to examine the moderating influence of parental education expectations on the relationship between perception of the “Double Reduction” policy and parental education anxiety.

### 3.4 Ethics statement

All research procedures involving human participants in this study adhered to the requirements of the local university’s ethics committee (Ethics Committee of the Research Center for Basic Education, Quality Inspection, and Evaluation, Shaanxi Normal University, Approval Number: 2022M13) and the Declaration of Helsinki of 1964. Prior consent was secured from all survey respondents during the data collection phase, and all gathered data were anonymized, ensuring the removal of any identifiers that could potentially link the data back to individuals.

## 4 Research findings

### 4.1 Common method bias test

Firstly, this study conducted procedural control in the design of the questionnaire, using measures such as reverse scoring to require respondents to submit their results anonymously; Secondly, Harman’s one-factor test (49) was utilized to assess common method biases in the collected questionnaire data. EFA was conducted, encompassing all items in the study, resulting in the extraction of five factors with eigenvalues greater than 1. Notably, the first factor accounted for 25.995% of the variance, which is below the critical threshold of 40% (50). Therefore, this study does not exhibit significant common method biases.

### 4.2 Descriptive statistics and correlation analysis

**Table 7** presents the detailed average scores and standard deviations of parental education anxiety, both overall and across its various dimensions. In this study, the theoretical mean of 3 is employed as the benchmark for assessing the degree of parental education anxiety. Specifically, parents with scores within the range of [0, 1] are considered to experience almost no anxiety; those with scores in the range of (1, 2] are classified as having mild anxiety; parents with scores in the range of (2, 3] are deemed to have moderate anxiety; those with scores in the range of (3, 4] are categorized as experiencing moderately high anxiety; and parents with scores in the range of (4, 5] are considered to have severe anxiety. The overall mean score of parental education anxiety stands at a high level of 3.70, indicating that parental education anxiety is prevalent among Chinese parents, with the degree of anxiety ranging from moderately high to severe. A comparison of the mean scores across the four internal dimensions of parental education anxiety reveals the following order of magnitude: Academic Attitude Anxiety > Educational Engagement Anxiety > School Selection Anxiety > Academic Performance Anxiety.

**Table 7:** Detailed breakdown of parental education anxiety (N = 2865).

Dimension	Synthesis	Academic performance	Academic attitudes	School selection	Educational engagement
M	3.70	3.43	3.91	3.63	3.84
SD	0.70	0.82	0.88	0.82	0.87

The mean values, standard deviations, and correlation coefficients among the core variables in this study are presented in **Table 8** below. The results indicate that family SES exhibits significant correlations with all other core variables, except for the variable of understanding of the “Double Reduction” policy. The variable of understanding of the “Double Reduction” policy shows a significant positive correlation with perception of the “Double Reduction” policy effectiveness, parental education anxiety, and parental education expectations. There is a notable positive relationship between perceived effectiveness of the “Double Reduction” policy and parental education anxiety, although the correlation with parental education expectations is not significant. Additionally, parental education anxiety demonstrates significant correlations with all the variables in question. These correlations among the core variables lay the groundwork for further research to be pursued in subsequent sections.

**Table 8:** Means, standard deviations and correlation coefficients of the main variables (N= 2865).

Core variable	Family SES	PEDRP	UDRP	PEA	PEE
Family SES	1				
PDRPE	0.021	1			
UDRP	-.069**	.620**	1		
PEA	-.041*	.239**	.155**	1	
PEE	.162**	.042*	.000	.062**	1
M	3.654	3.470	3.298	3.703	6.440
SD	1.096	0.796	0.876	0.703	0.888

* p<0.05, ** p<0.01; (two-tailed test). family SES, family socio-economic status; PDRPE, perception of the “Double Reduction” policy effectiveness; UDRP, Understanding of the “Double Reduction” Policy; PEA, Parental Education Anxiety; PEE, Parental Education Expectations.

### 4.3 Testing the mediating effect of parents’ perception of the “Double Reduction” policy on the influence of family SES on parental education anxiety

Prior to exploring the mediating role of perceived the “Double Reduction” policy in the relationship between family SES and parental education anxiety, an initial assessment was conducted on the direct effect of family SES on parental education anxiety. The outcomes of this assessment are summarized in **Table 9** below. Model 1, serving as the benchmark, incorporates controls for pertinent variables. The results demonstrate a statistically significant negative correlation between family SES and parental education anxiety (β = -0.062, p < 0.001), suggesting that a one-unit increment in family SES is associated with a notable 0.062-point decrease in parental education anxiety. In essence, the coefficient c in Equation 1 is found to be significant, thereby validating Hypothesis 1. Based on Model 1, Model 2 and Model 3 incorporate the perception of the “Double Reduction” policy effectiveness and the understanding of the “Double Reduction” policy, respectively. As shown in **Table 7**, the mediator variables for these two dimensions have significant positive effects on parental education anxiety, indicating that the coefficients b for the mediator variables in both dimensions in Equation 3 are statistically significant. Among these mediator variables, the perceived effectiveness of the “Double Reduction” policy has the most pronounced impact, changing the regression coefficient of family SES in the regression model from -0.062 to -0.052. This suggests that, among the two dimensions’ mediating effects, the perception of the “Double Reduction” policy effectiveness exerts a greater mediating effect.

**Table 9:** Regression analysis of family SES affecting parental education anxiety (N= 2865).

Variables	Equation 1		Equation 3			
	Model 1		Model 2		Model 3	
	factor	SE	factor	SE	factor	SE
1	3.695***	0.151	3.029***	0.155	3.27***	0.157
2	-0.001	0.031	0.002	0.03	0.008	0.03
3	-0.028	0.022	-0.027	0.021	-0.032*	0.022
4	-0.018	0.015	-0.022	0.015	-0.021	0.015
5	-0.035*	0.026	-0.036*	0.026	-0.036*	0.026
6	0.07***	0.015	0.06***	0.015	0.067***	0.015
7	-0.062***	0.014	-0.067***	0.013	-0.052**	0.013
8			0.238***	0.016		
9					0.154***	0.015
R²	0.009		0.065			

* p<0.05, ** p<0.01, ***p<0.001; (two-tailed test). Variables in the model are standardized and brought into the regression equation; 1:constant; 2: genders; 3: age; 4: political profile; 5: Sex of children; 6: Parental educational expectations (PEE); 7: Family SES; 8: perception of the “Double Reduction” policy effectiveness (PDRPE); 9: Understanding of the “Double Reduction” policy (UDRP).

The regression results for Equation 2 are detailed in **Table 10**. with relevant control variables held constant, there exists a significant negative relationship between family SES and the perception of the “Double Reduction” policy effectiveness, indicating that the coefficient a in Equation 2 is statistically significant. According to the principles of the stepwise testing method, when both coefficients a and b are significant, the mediating effect is deemed established, and in such cases, the power of the stepwise method surpasses that of the Bootstrap method, thereby obviating the need for further mediation testing using the Bootstrap approach. Furthermore, the coefficient c’ derived from Equation 3 with the inclusion of the mediator variable, namely the perception of the “Double Reduction” policy effectiveness, is also significant, suggesting that perception of the “Double Reduction” policy effectiveness partially mediates the impact of family SES on parental education anxiety. However, the Bootstrap method is particularly robust for small samples or non-normal data. The parametric percentile Bootstrap method outperforms traditional methods (51). Therefore, this study continues to use the parametric percentile Bootstrap method to verify the results of the stepwise method. For specific content, see **Table 11** below. The results show that the 95% confidence interval of the mediating effect is [-0.018, -0.003], which does not include zero, further indicating the existence of the mediating effect.

**Table 10:** Effects of family SES on mediating variables (N= 2865).

Variable or dimension	Equation 2			
	UDRP		PDRPE	
	factor	SE	factor	SE
constant	3.176	0.171	3.445	0.188
Family SES	0.02	0.015	-0.068***	0.017
control variables	control		control	
R²	0.002		0.009	

* p<0.05, ** p<0.01, ***p<0.001; (two-tailed test). Variables in the model are standardized and brought into the regression equation, UDRP, Understanding of the “Double Reduction” policy (UDRP); PDRPE, Perception of the “Double Reduction” policy effectiveness.

**Table 11:** Effects of family SES on mediating variables (N= 2865).

Path 1: Family SES--UDRP--PEA					
Effect Classification	Factor	SE	Percentile 95% CI		
			Lower	Upper	Proportion of Effect/%
Total effect	-0.027	0.012	-0.050	-0.003	
Direct effect	-0.030	0.012	-0.053	-0.007	
mediating effect	0.003	0.003	-0.003	0.009	No intermediary effect
Path 2: Family SES--PDRPE--PEA					
Effect Classification	Factor	SE	Percentile 95% CI		
			Lower	Upper	Proportion of Effect/%
Total effect	-0.044	0.014	-0.067	-0.014	
Direct effect	-0.034	0.013	-0.060	-0.008	77.27
mediating effect	-0.010	0.004	-0.018	-0.003	22.73

bootstrapping, 5000; UDRP, Understanding of the “Double Reduction” policy (UDRP); PDRPE, Perception of the “Double Reduction” policy effectiveness; PEA, Parental Education Anxiety.

On the other hand, the relationship between family SES and the understanding of the “Double Reduction” policy is insignificant, as indicated by the nonsignificant coefficient a in Equation 2. According to the principles of the stepwise regression method, when at least one of the coefficients (a or b) is nonsignificant, further mediation analysis using the Bootstrap method is necessary, as shown in **Table 11** below. The Bootstrap results reveal that the mediation effect of the understanding of the “Double Reduction” policy on the influence of family SES on parental education anxiety has a 95% confidence interval spanning from -0.0056 to 0.0150, which encompasses zero. This finding suggests that the understanding of the “Double Reduction” policy does not serve as a mediator in the relationship between family SES and parental education anxiety. In conclusion, Hypothesis 2 is partially confirmed.

Within the intermediary variable of perception of the “Double Reduction” policy effectiveness, specifically focusing on the dimension of perception of the “Double Reduction” policy effectiveness that serves as a mediator, the total effect, direct effect, mediating effect, and the proportion of the mediating effect within the total effect for this dimension are presented in **Table 11**. The findings indicate that the overall effect of family SES on parental education anxiety is -0.044. Furthermore, the mediating effect of the perception of the “Double Reduction” policy effectiveness dimension accounts for 22.73% of the total effect, thereby confirming Hypotheses 3 and 4 proposed in this study.

### 4.4 Texting the moderating effect of parental education expectations between perception of the “Double Reduction” policy effectiveness and parental education anxiety

To validate the preceding hypotheses, this study further analyzes the moderating role of parental education expectations between the perception of the “Double Reduction” policy effectiveness and parental education anxiety. The analytical results are presented in **Table 12** below. The findings reveal that, upon incorporating parental education expectations into the model, parental education expectations positively predict parental education anxiety (β = 0.053, t = 3.572, p < 0.001). Notably, the interaction between the perception of the “Double Reduction” policy effectiveness and parental education expectations significantly predicts PEA (β = -0.079, t = -4.682, p < 0.001), suggesting that parental education expectations moderate the predictive role of the perceived effectiveness of the “Double Reduction” policy on parental education anxiety. Consequently, Hypothesis 6 is confirmed.

**Table 12:** Mediation model test with moderation (N = 2865).

Outcome variable	Predictor variable	Standardized regression coefficient	SE	t	95% confidence interval	
					LLCI	ULCI
Parental Education Anxiety	1	4.051	0.123	32.931***	3.81	4.292
	2	-0.037	0.013	-2.738**	-0.063	-0.01
	3	0.126	0.015	8.486***	0.097	0.155
	4	0.053	0.015	3.572***	0.024	0.082
	3*4	-0.079	0.017	-4.682***	-0.113	-0.046
	control variables	control				
R²		0.007				
F²		21.92				

*p < 0.05, **p < 0.01, ***p < 0.001; (two-tailed test). 1: Intercept term (constant); 2: Family SES; 3: perception of the “Double Reduction” policy effectiveness (PDRPE); 4: Parental education expectations (PEE).

**Figure 2:**
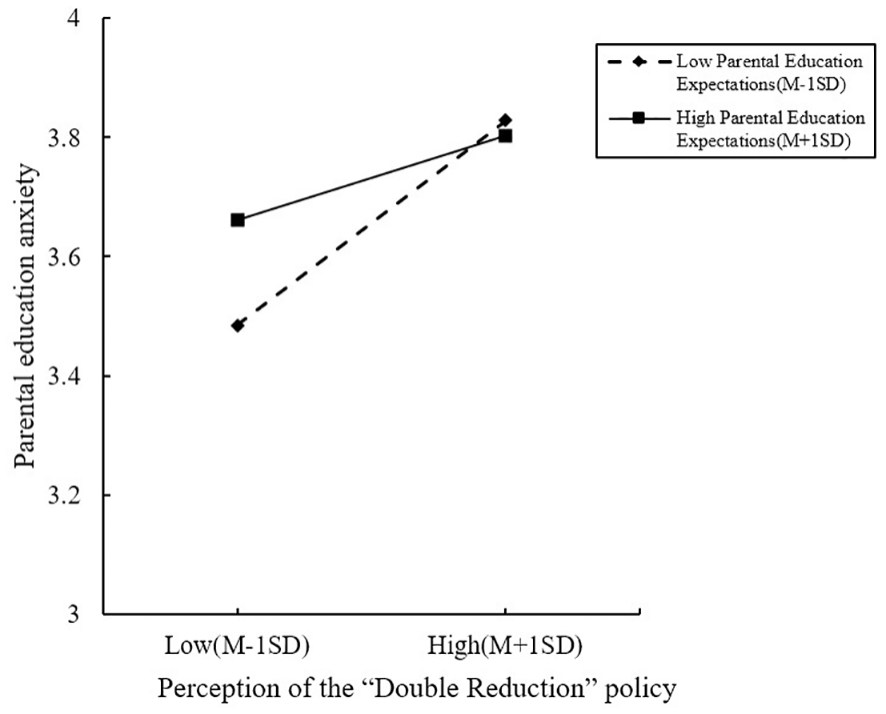
The moderating role of parental education expectations between perception of the "Double Reduction" policy and parental education anxiety.

**Figure 3:**
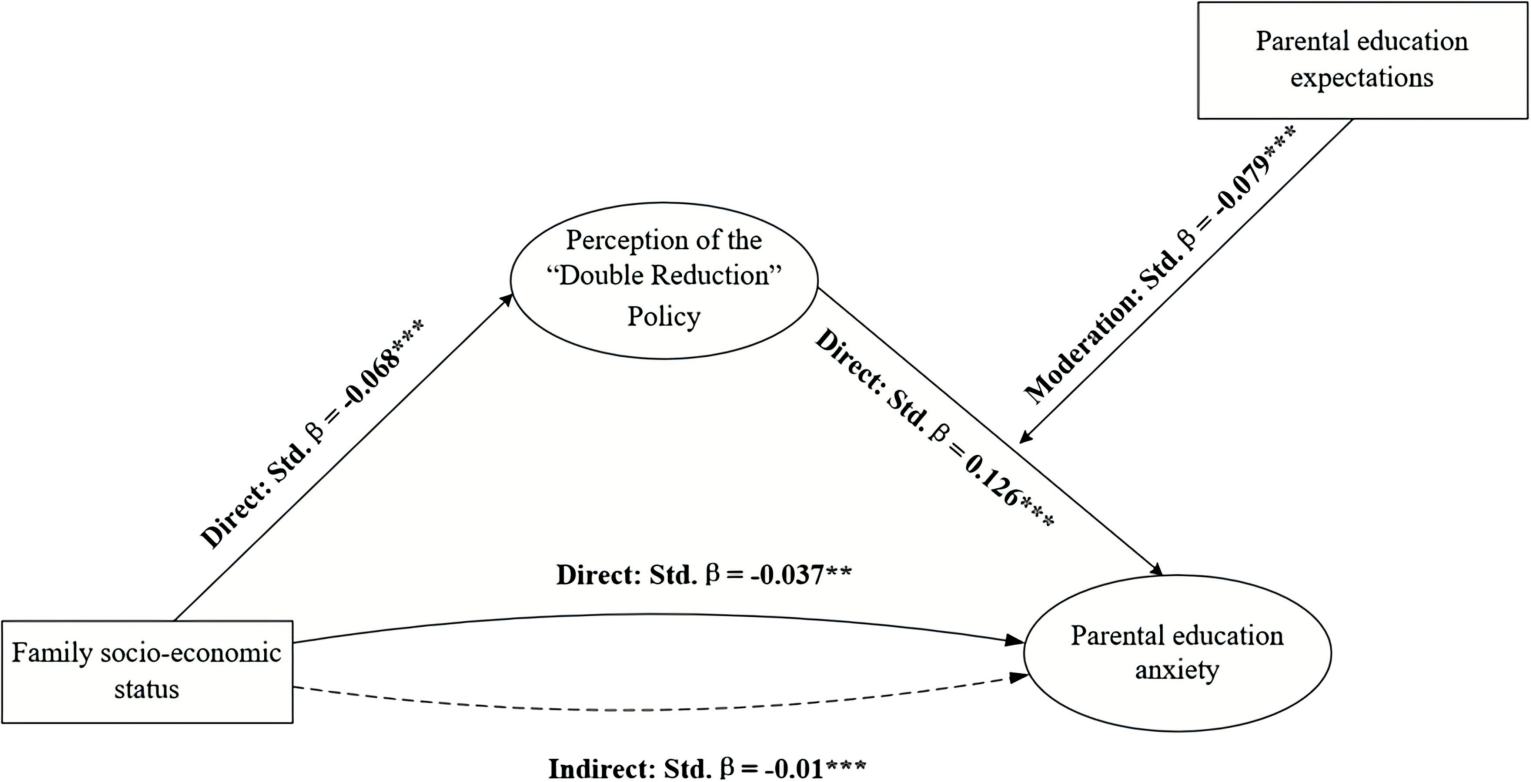
Parameter estimates of the mechanisms of formation of parental education anxiety.

To further explore the essence of the moderating effect, following scientist’ advice (52), a method involving one standard deviation above and below the mean was adopted to categorize parental education expectations into high and low groups for the construction of simple slope plots. The results are illustrated in **Figure 2** below. For both the low and high educational expectation groups, there was a significant positive predictive trend observed between perception of the “Double Reduction” policy effectiveness and parental education anxiety. However, the perception of the “Double Reduction” policy effectiveness on educational anxiety was stronger among parents in the low educational expectations group (β=0.196, t=9.143, p<0.001) compared to those in the high educational expectation group (β=0.081, t=4.650, p<0.001). This indicates that the adverse impact of the perception of the “Double Reduction” policy effectiveness on parental education anxiety is more pronounced when educational expectations are lower. In other words, as parental education expectations increase, the perception of the “Double Reduction” policy effectiveness is less inclined to manifest as educational anxiety. Consequently, a moderated mediation model depicting the influence of family SES on parental education anxiety, along with its parameter estimates, is derived and presented in **Figure 3** below.

## 5 Discussion

### 5.1 Educational anxiety of Chinese parents remains moderate to severe under the “Double Reduction” policy background

On one hand, from a holistic perspective, parental education anxiety remains extremely prevalent in the context of the “Double Reduction” policy. Nearly all parents participating in this study have expressed varying degrees of educational anxiety, indicating that, after three years of implementation, the policy has yet to fully achieve its intended goals. Moreover, there may exist a paradox where the enactment of the “Double Reduction” policy paradoxically increases parental education anxiety. This study further corroborates scientists’ perspectives (53), yet it contrasts with previous research that suggested the introduction of the policy could effectively alleviate parental education anxiety (54). On the other hand, in terms of the specific dimensions of parental education anxiety, parents’ emotional anxiety concerning their children’s academic attitudes ranks foremost, while the previously heightened focus on outcome-oriented anxiety related to academic performance (55) now falls to the bottom. This shift could be attributed to the “Double Reduction” policy’s requirement for schools to implement a practice of “not disclosing specific exam scores but only announcing students’ exam grade levels,” effectively shielding parents’ concerns and anxieties about academic performance from direct scrutiny. However, in the absence of timely incentives derived from grades, students may exhibit a decline in their competitive spirit and a relaxation in their learning attitudes. Consequently, parents have shifted their anxiety from academic performance to academic attitudes.

### 5.2 The influence of family socio-economic status on parental education anxiety under the “Double Reduction” policy background

Family SES negatively influences parental education anxiety, meaning that parents with family SES are less prone to feeling anxious. This is primarily because family SES is often viewed as one indicator of a family’s educational capital. The level of family SES directly impacts a family’s ability to invest in education. Parents perceive children’s educational expenses as investments in the reproduction of family human capital (56). Parents with family SES will allocate more resources to provide their children with opportunities for supplementary education outside of school. More notably, the “Double Reduction” policy’s radical approach to eliminating off-campus tutoring institutions has led to a surge in private tutoring prices (57). This phenomenon is not unique to China. The educational competition landscapes in China and South Korea exhibit striking convergence. South Korea’s private tutoring system, established since the 1960s, has become deeply embedded in its social stratification mechanisms. The frequency and quality of students’ extracurricular tutoring participation demonstrate a positive correlation with family SES. A 2023 survey (58) revealed that South Korean middle school students spend an average of 596,000 KRW monthly (approximately 3,200 CNY) on supplementary education. The proliferation of private tutoring has essentially erected new educational access barriers. Families with superior family SES maintain competitive advantages through premium tutoring services (59), while resource-constrained households endure dual pressures: structural deprivation of educational opportunities for their children and parenting guilt stemming from resource scarcity. This dual burden significantly intensifies parental education anxiety.

### 5.3 The influence of perception of the “Double Reduction” policy on family Socio-economic status and parental education anxiety

Family SES directly influences parental education anxiety and also indirectly affects it through parents’ perception of the “Double Reduction” policy. Specifically, on the on hand, the dimension of perception of the “Double Reduction” policy effectiveness within parents’ perception of the “Double Reduction” policy acts as a partial mediator between family SES and parental education anxiety, accounting for 22.73% of the mediation effect. Notably, parents’ perception of the “Double Reduction” policy effectiveness demonstrates a positive association with parental education anxiety, this ostensibly counterintuitive phenomenon reveals a fundamental paradox between the policy’s equity-oriented(original intent) and its practical outcomes within China’s exam-driven, resource-scarce educational system. This counterintuitive phenomenon finds robust theoretical support in Conservation of Resources theory: Parents with heightened the “Double Reduction” policy effectiveness awareness demonstrate earlier recognition of the irreversible depletion of conventional educational resources (particularly shadow education services), and acute consciousness regarding the compensatory limitations of family capital (encompassing tutoring capacity, social networks, and economic resources) under the “Double Reduction” policy constraints. The structural asymmetry between resource attrition and compensatory capacity induces psychological imbalance, thereby escalating parental education anxiety.

On the other hand, Notably diverging from prior investigations conducted during the initial implementation phase of the “Double Reduction” policy (2021-2022) (60), our study reveals that parental understanding of the “Double Reduction” policy does not demonstrate a statistically significant mediating role in the family SES and parental education anxiety relationship. This finding suggests the need to reassess the role of family SES throughout the policy lifecycle: In the early stages of the “Double Reduction” policy implementation, parents from higher SES backgrounds, leveraging their rich cultural capital and extensive social networks, were able to quickly decode policy signals and effectively adjust their educational strategies to mitigate the anxiety caused by the abrupt policy changes. However, as the “Double Reduction” policy has entered a stable phase, the government and schools have employed multi-level, multi-channel dissemination, leading to a gradual shift in parents’ understanding of the policy from “individualized interpretation” to “collective consensus”. During this process, the cultural capital advantage of high-SES families has been gradually diminished, and the cognitive gap for low-SES families has been narrowed. Therefore, the results of this study indicate that the understanding of the “Double Reduction” policy is not a mediating factor in the relationship between family SES and parental educational anxiety.

### 5.4 The influence of parental education expectations on the perception of the “Double Reduction” policy effectiveness and parental education anxiety

In the process of data analysis, the interaction between family SES and parental education expectations failed to significantly influence parents’ perception of the “Double Reduction” policy effectiveness and their educational anxiety. However, the interaction between parents’ perception of the “Double Reduction” policy effectiveness and educational expectations had a notable negative effect on parental education anxiety, suggesting that educational expectations moderate the impact of family SES on parental education anxiety. In other words, higher parental education expectations attenuate the positive association between parents’ perception of the “Double Reduction” policy and their educational anxiety. This confirms Hypothesis 6 in our study.

On one hand, the findings of this study underscore a paradox in policy implementation: the better parents’ perception of the “Double Reduction” policy effectiveness, the greater their anxiety. The effective implementation of the policy implies a tangible reduction in both in-school and out-of-school burdens for students. However, the persistent and intense competition sends a signal to parents that their children may lose academic competitiveness if they rigidly adhere to the government’s policy while their competitors are using other means to enhance their abilities (61). On the other hand, the results of this study provide an explanation for the persistence of shadow education in the context of the “Double Reduction” policy. Driven by the competitive atmosphere and the fear of “falling behind”, parents are caught in a “theater effect” and “herd effect” of passive competition and blind participation, which significantly fuels the popularity of extracurricular academic training and stimulates various latent demands for tutoring. Parents with high educational expectations, concerned that their children may fall behind due to the policy’s reduction of burdens, are willing to invest heavily in seeking out-of-school tutoring. In this manner, parents adopt a costly and misguided approach to managing their educational anxiety.

## 6 Implication

This study does not aim to critique the “Double Reduction” policy, but rather seeks to adopt the parental perspective as a lens for understanding the policy, thereby informing the direction for its further elaboration in the future.

Firstly, the resources available to families exercise a significant influence on their children’s access to social opportunities and the distribution of educational resources, potentially exacerbating educational inequality (62). Ensuring the public welfare and fairness of education is a fundamental educational responsibility of modern governments. Simply curbing extracurricular tutoring and drastically reducing student workloads cannot fundamentally alleviate parental educational anxiety. We must confront the impact of family SES on such anxieties and formulate compensatory policies to narrow educational disparities across family SES groups. This requires substantial increases in educational investments for under-resourced regions and disadvantaged populations, coupled with expanded free school services to holistically enhance regional educational quality. International precedents offer valuable insights, such as Germany’s (63) school scholarship system that ensures equitable access to premium educational resources regardless of social stratification. China could further strengthen welfare-oriented education through context-specific innovations, including establishing dynamic identification mechanisms to accurately target economically vulnerable students and families, paired with tailored support like need-based grants and tuition exemptions. These measures not only alleviate financial burdens on disadvantaged households but also create enhanced developmental opportunities for marginalized students, thereby advancing educational equity.

Secondly, drawing upon multi-dimensional evaluation models from abroad, China should expedite the reform of its current educational assessment system, thereby truly liberating students from the oppressive burden of excessive examinations. For example, in the past decade, the majority of U.S. states have implemented a multi-dimensional and evidence-based assessment framework tailored for primary and secondary students (64). Additionally, PISA 2025 innovates its evaluation paradigm to align with the demands of the digital era (65), while TIMSS 2019 employs computer-assisted assessment techniques to address the complexities involved in evaluating students (66).

Thirdly, It is crucial to adopt a dialectical perspective on the positive role of homework in knowledge consolidation while implementing scientific governance of off-campus tutoring institutions to achieve dynamic equilibrium between regulating market order and fulfilling educational functions. As a society deeply rooted in Confucian educational traditions like China, Singapore also faces challenges stemming from the proliferation of private tutoring due to high-intensity educational competition. Consequently, Singapore’s governance strategies hold significant referential value: On the one hand, to enhance the scientific management of private tutoring institutions, the Singaporean government mandates registration with the Ministry of Education for all organizations providing off-campus training to over 10 students, accompanied by stringent requirements for facilities (e.g., training venues, fire safety standards) (67). On the other hand, leveraging public school resources, the Singaporean government enriches students’ campus experiences and elevates educational quality through inquiry-based and practice-oriented teaching activities, such as interdisciplinary projects and community service learning programs (68).

Fourthly, parents’ excessively high educational expectations can readily exacerbate their educational anxiety. To alleviate this, parents must initiate a proactive transformation of their educational beliefs, progressively dismantling biases against vocational education, rectifying blind admiration for general education, and overcoming the tendency to prioritize academics over technical skills. Instead, they should empower their children to choose paths based on their interests and expertise, and adjust their educational expectations from multiple perspectives and through various channels, aligning them with their children’s potential, interests, and academic performance.

## 7 Limitations and future research

First, the cross-sectional design constrains causal inference regarding the relationships between policy perceptions and educational anxiety. While this approach was necessitated by resource constraints and the need for timely evaluation of the “Double Reduction” policy, longitudinal data tracking changes in parental attitudes over time would provide stronger causal evidence. Second, this study collects data through parent-reported questionnaires. Although strict anonymity was implemented during the data pre-processing stage and the analysis indicates no significant self-reporting bias, there remains a potential risk of bias arising from social desirability in policy perception responses. Moreover, the exclusive reliance on parental reports, without incorporating child-level data such as standardized academic performance, somewhat limits the explanatory power of the relationships between variables. Third, the political identity attributes of the interviewed parents in this study may influence their perceptions and evaluations of the “Double Reduction” policy. Some respondents—especially non-Party members—might have felt freer to criticize the policy, anticipating reforms. Conversely, others—particularly Party members—may have perceived criticizing a moderately successful policy as politically safer than endorsing it.

## Data Availability

The raw data supporting the conclusions of this article will be made available by the authors, without undue reservation.
